# Hybrid Structure Multichannel All-Fiber Current Sensor

**DOI:** 10.3390/s17081770

**Published:** 2017-08-02

**Authors:** Junzhen Jiang, Hao Zhang, Youwu He, Yishen Qiu

**Affiliations:** 1 Key Laboratory of OptoElectronic Science and Technology for Medicine of Ministry of Education, Fujian Provincial Key Laboratory of Photonics Technology, Fujian Normal University, Fuzhou 350007, China; jzjiang@fjnu.edu.cn (J.J.); ywhe@fjnu.edu.cn (Y.H.); 2 Department of Electronic Information Science, Fujian Jiangxia College, Fuzhou 350007, China; 1698091502181@fjjxu.edu.cn

**Keywords:** fiber optics, current sensors, hybrid structure, multi-channel, time division multiplexing

## Abstract

We have experimentally developed a hybrid-structure multi-channel all-fiber current sensor with ordinary silica fiber using fiber loop architecture. According to the rationale of time division multiplexing, the sensor combines parallel and serial structures. The purpose of the hybrid-structure multi-channel all-fiber current sensor is to get more information from the different measured points simultaneously. In addition, the hybrid-structure fiber current sensor exhibited a good linear response for each channel. A three-channel experiment was performed in the study and showed that the system could detect different current positions. Each channel could individually detect the current and needed a separate calibration system. Furthermore, the three channels will not affect each other.

## 1. Introduction

All-fiber optic current sensors (AFCSs) are mainly based on the Faraday effects in the coil of an optical fiber around a current conductor and have attracted significant interest in recent years owing to their immunity to compact design, fast response time, electromagnetic interference, and so on [1,2,3,4,5,6]. However, a disadvantage of the AFCS is its limited sensitivity to the magnetic field. Sensitivity is decided by the Verdet constant of silica material itself and linear birefringence induced by bending and can be enhanced by the use of either a very long fiber or a doped fiber. However, these methods can increase the size, are high in cost, and are more sensitive to temperature and the introduction of birefringence [5,6]. Therefore, fiber loops utilizing ring-down architecture have wide applications in many aspects, such as sensors, lasers, and so on [7,8,9,10] and have advantages of better flexibility, lower cost, and stronger extensibility [7,8,9,10,11,12,13]. In optical current sensing applications, the fiber loop can improve sensitivity by increasing the effective interactive length between the optical fiber and magnetic field [11].

In 2012, we reported an all fiber current sensor which had high sensitivity based on fiber loop structure. In the AFCS, it can increase sensitivity which the signal can repeat through the sensor head until the light intensity attenuation in full. However, the aforementioned architecture only detects one channel current [14,15]. Sometimes, it is necessary for people to measure current at different points. For example, in measuring of different locations in the power grid, or in measuring of the three-phase electricity, it needs the current monitoring at the difference points. If every point needs a set of sensors—including one pulsed laser, one sensor head, one detector, and one digital signal processing system—it would be very expensive. Recently, we proposed a parallel-structure dual-channel AFCS and a serial-structure dual-channel AFCS [16]. The parallel structure exhibited a good light isolation effect and low light utilization efficiency. On the contrary, the serial structure exhibited a poor light isolation effect and high light utilization efficiency. Neither structure could achieve a satisfactory performance. Subsequently, to achieve current monitoring of the transmission lines, we developed a hybrid-structure multi-channel AFCS, which was a combination of the serial and parallel structures.

Compared with the individual serial and parallel structures, the hybrid structure simultaneously exhibited a good light isolation effect and good light utilization efficiency as a hybrid-structure three-channel system only needed two fiber delay coils. Based on time division multiplexing, we can obtain more information from the sensor using the hybrid structure since there is only one system and three sensor heads can complete current detection for the three channels. A hybrid-structure sensor has the advantages of better flexibility, smaller size, lower costs, and greater strength.

## 2. Materials and Methods

**Figure 1:**
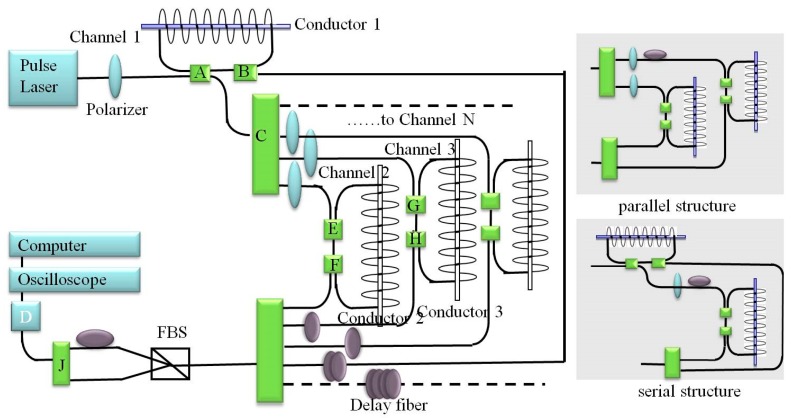
Configuration of the hybrid-structure multichannel all-fiber current sensor.

Figure 1 (left) shows the configuration of the hybrid-structure multi-channel all-fiber current sensor and Figure 1 (right) shows the serial and parallel structures. The two structures are all based on the rationale of time division multiplexing. A fiber solenoid was used as a current sensor head in the fiber loop utilizing ring-down structure. The fiber loop structure was composed of two fiber couplers and a fiber solenoid. The optical signal could repeat through the sensor head until the light intensity attenuation was full, increasing the Faraday rotation angle and enhancing the current sensitivity correspondingly. The optical pulses received from the 2 × 2 coupler were partly coupled into the first fiber loop structure and partly split into *N* ways using a 1 × *N* fiber coupler. Subsequently, the pulses from the 1 × *N* fiber coupler were coupled into the second fiber loop structures, the third fiber loop structures the *N* fiber loop structures, respectively. Each split pulse was forwarded into linearly polarized light through the polarizer. Subsequently, these linearly polarized light pulses were coupled into the fiber loops utilizing ring-down architecture and circulated inside the architecture repeatedly before full light intensity attenuation. At the receiver, the *N*-way pulses light signals were combined into the polarizing beam splitter (PBS) using an *N* × 1 fiber coupler. The *N*-way fiber ring-down pulse light of the sensors was combined based on time division multiplexing by fiber-delay coils. Two beams of orthogonally polarized lights were output from the PBS. A delay fiber coil was inserted behind the PBS to avoid the interference between the two orthogonal polarization lights. The two orthogonally polarized beams were combined into one fiber by the 2 × 1 coupler and the final results were measured by a photodetector (D).

The experimental layout of the three-channel fiber current sensor used in our experiment was made up of a 1550 nm pulsed laser (with peak power 10 W, average output power 3.8 mW, output frequency 1 MHz, and pulse width 15 ns), one 2 × 2 fiber coupler, one 3 × 1 fiber coupler, one PBS (1 × 2, 1550 nm), one detector, three fiber solenoids, three polarizers, seven 2 × 1 fiber couplers, and one digital signal processing system. The three fiber sensor solenoids were comprised of a standard single mode fiber with diameter of 100 mm and 200 turns. Channel 1 was made up of a fiber solenoid and couplers A and B. Channel 2 was made up of a fiber solenoid and couplers E and F. Channel 3 was made up of a fiber solenoid and couplers G and H. The splitting ratios (in parentheses) of the couplers were A (3:97); E and G (5:95); B, F and H (1:99); C and J (50:50); I (33.3:33.3:33.3). Light from the laser went through the 97% port of coupler A and input into the fiber loop architecture. The pulse travelling through the other 97% port of coupler A was split into two by the coupler C. Each split pulse went through the 5% port of the coupler E/G and input into the fiber loop architecture. In each cycle, a little part of the pulse light input into the 1% port of the coupler B/F/H. The light through the coupler B/F/H were combined into the PBS using coupler I. Two beams of orthogonally polarized lights output through the PBS were combined into one fiber by coupler J. The final lights through coupler J were probed by the photodetector (D) and the digital processing system. The direct current (DC) ranged from 0 to 1000 A in the experiment for Channels 1 and 2. The direct current (DC) ranged from 0 to 2000 A in the experiment for Channel 3.

Some delay fiber coils were inserted on the back of coupler C and the PBS to avoid the mutual interference between the channel signals and between the two beams of orthogonal polarization lights. To achieve non-interference between two beams of orthogonal polarization lights, the width of the two beams of orthogonal polarization lights should be greater than or equal to the width of the laser pulse base on the time-division multiplexing (TDM). The length of the delay fiber coil inserted behind the PBS should be greater or equal to

*L_O_* = (*c*/*n*) × *t_l_* (1)

where *t_l_* is the width of the laser pulse. According to Equation (1), a delay line (of length *L_O_* = 3 m) was inserted behind the PBS.

**Figure 2:**
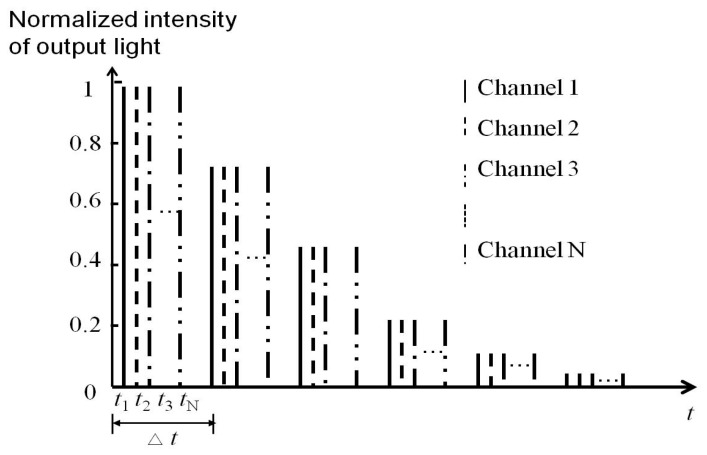
Simulated signals for the multichannel fiber current sensor.

The simulation signals of the hybrid-structure fiber current sensor are illustrated in Figure 2, where the *x*-axis is time and the *y*-axis is normalized intensity, where ∆*t* = *t*_1_ − *t* is the round-trip time of the laser pulse in the fiber loop [7]. Furthermore, ∆*t* is determined by the length *L_H_* of the fiber sensor head with a diameter of 100 mm and 200 turns. To achieve non-interference between the signals of the three channels, the length of the delay fiber coil was inserted on the front of the loop and was selected based on

*L_D_* (*N*) = (*N* − 1) × *L_H_*/*N*_max_ (2)

The experiment utilized a hybrid-structure three-channel fiber current sensor to detect three different current positions. Furthermore, *N* is the channel number and *N*_max_ = 3, the length of delay fiber is 21 m for *N* = 2, and the length is 42 m for *N* = 3.

## 3. Results

**Figure 3:**
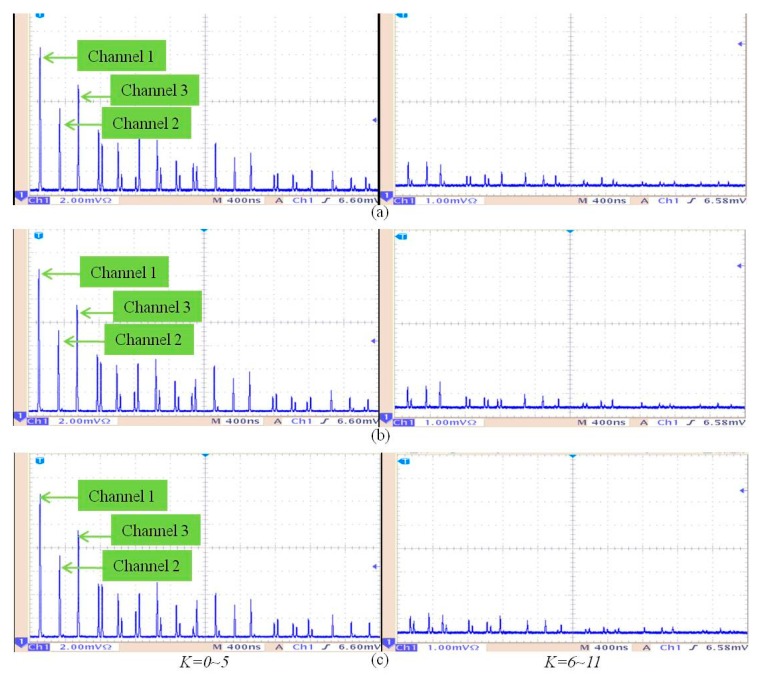
Intensity variations of light pulses taken from a Tektronix TDS3054B 500 digital oscilloscope (Tektronix, Beaverton, WA, USA): (**a**) three channels for currents of 0 A, respectively; (**b**) three channels for currents of 500 A, 500 A, and 1000 A, respectively; (**c**) three channels for currents of 1000 A, 1000 A, and 2000 A, respectively.

Figure 3 shows the intensity changes of the three-channels when the currents for 0, 500, 1000 A via Channel 1 and 2 and the currents of 0, 1000, 2000 A via Channel 3, respectively. Figure 3 shows the time scale for 400 ns. To the left of Figure 3, the number *K* of round-trip signals is from zero to five. To the right of Figure 3, *K* is from 6 to 11. Figure 3a depicts light intensity changes the three-channels when currents of 0 A are registered via Channels 1, 2, and 3, separately. Figure 3b depicts the light intensity changes of the three-channels when currents of 500 A are registered via Channels 1 and 2, and when a current of 1000 A is registered via Channel 3, separately. Figure 3c depicts the light intensity changes of the three-channels when currents of 1000 A are registered via Channels 1 and 2, and when a current of 2000 A is registered via Channel 3, separately. When *K* is 0, the waveforms do not undergo a round trip in the fiber loop structure. When *K* is 1, the waveforms undergo one round trip in the fiber loop structure, etc. ${{\left|E\right|}^{2}}_{⊥}$ and ${{\left|E\right|}^{2}}_{∥}$ in Equation (3) are the light intensities of the adjacent pairs of peaks, respectively. The polarization degree of the export pulse [17] is

(3) $$P=\frac{{{\left|E\right|}^{2}}_{⊥}-{{\left|E\right|}^{2}}_{∥}}{{{\left|E\right|}^{2}}_{⊥}+{{\left|E\right|}^{2}}_{∥}}$$

The variation of the polarization degrees ∆*P* is the change of the polarization degrees in two kinds of states for the current-off and current-on. The expression is

(4) $$\Delta P=P-P_{0}$$

**Figure 4:**
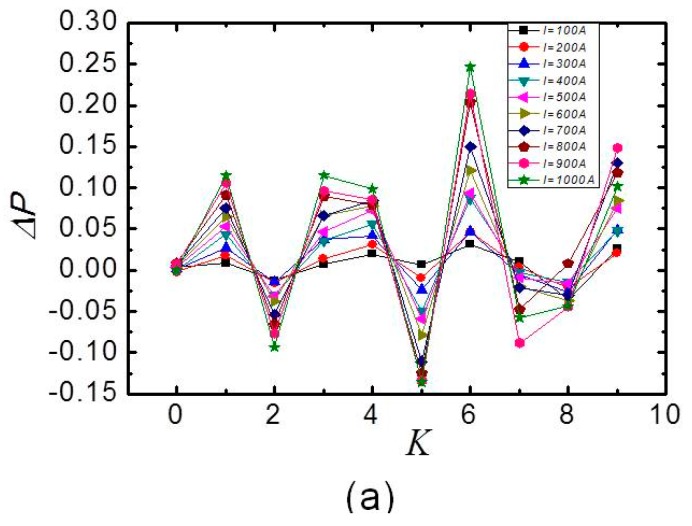

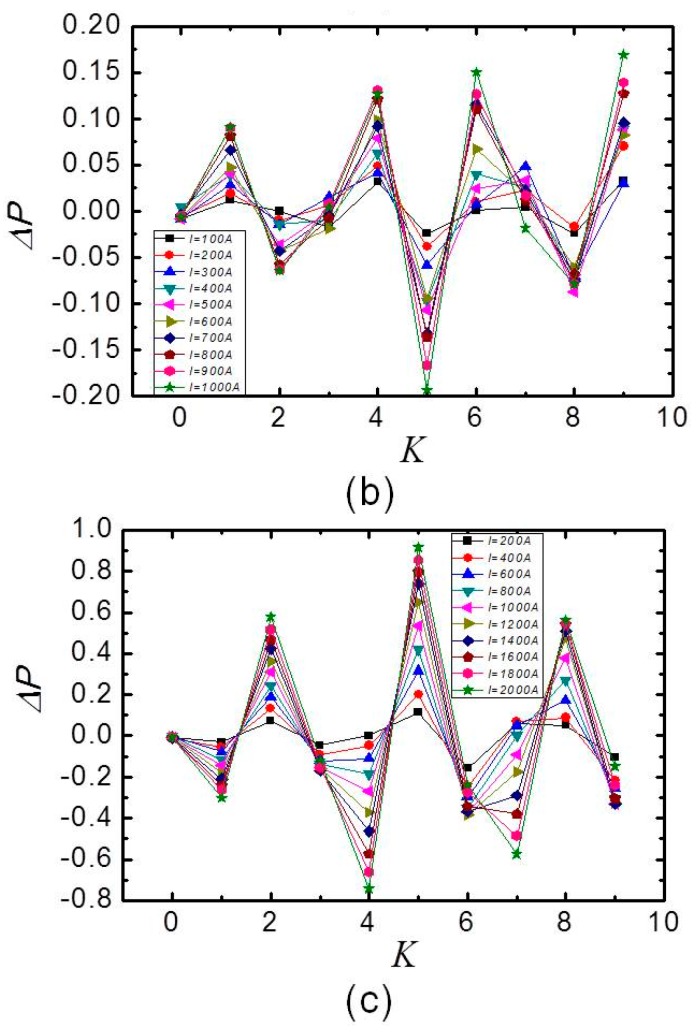
Variation in the output signal ∆*P* versus *K* for (**a**) Channel 1; (**b**) Channel 2; and (**c**) Channel 3.

The figure displays the three channels’ intensity variation of two adjacent peaks change with the *K*. The variation of the polarization degree, i.e., the normalized power transmitted by the linear polarizer, against *K* can be obtained from the oscilloscope data. The final results are displayed in Figure 4.

## 4. Discussion

The relationship of variation in the degree of polarization ∆*P* with *K* can be obtained from the oscilloscope data, and the results are shown in Figure 4. Figure 4a–c show the results of Channels 1, 2, and 3, respectively. Figure 4a,b shows the relationship when the current *I* = 100 A, 200 A, 300 A, …, 1000 A. Figure 4c shows the relationship when the current *I* = 200 A, 400 A, 600 A, …, 2000 A. It was evident that ∆*P* oscillation increased with *K*, but the decay waveform contour of the light was fitted to a single exponential decay for each fiber loop, and the intensity of the optical signals of the three channels was also the exponential decay with the increase of *K*. Therefore, the variation volatility of the polarization state increased and the attenuation of light with the increase in *K* should be considered in the selection of the best *K*.

**Figure 5:**
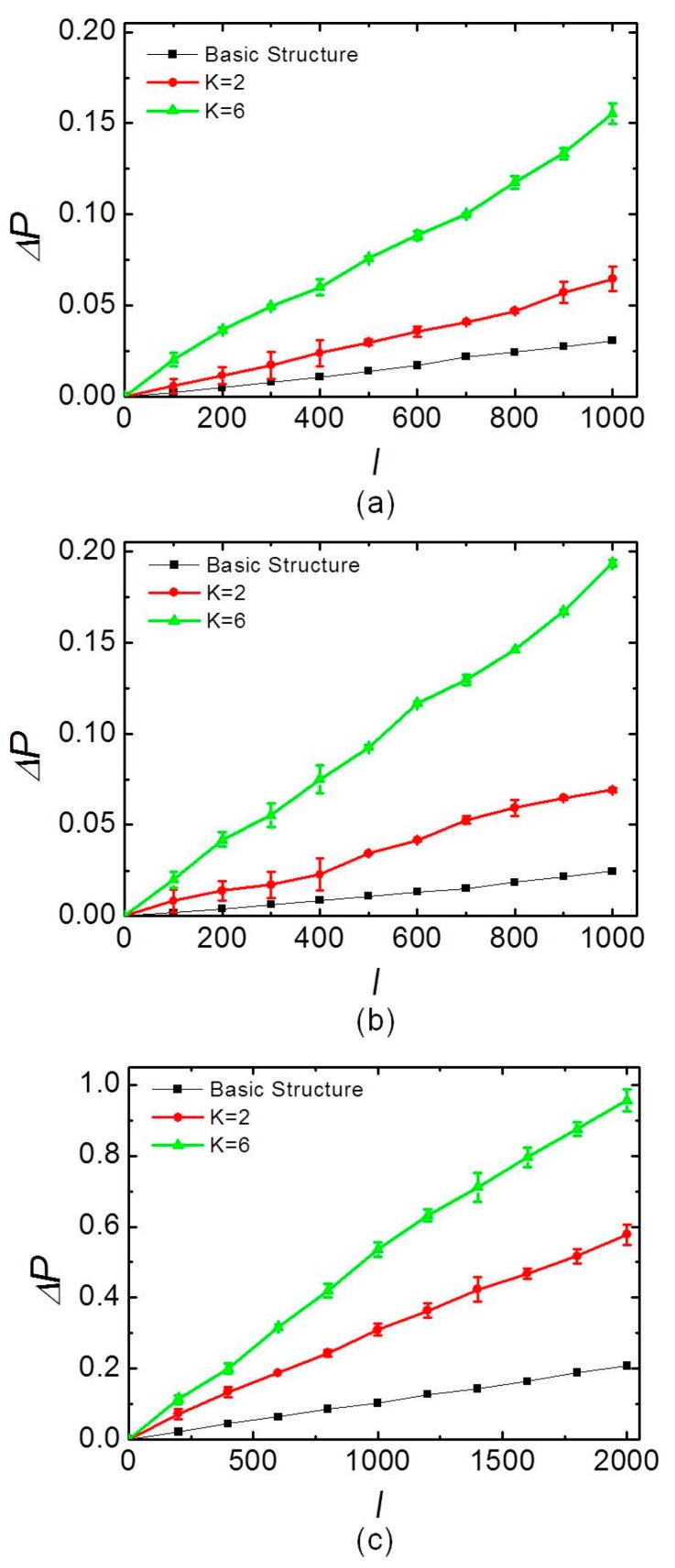
Relationship between the output signal ∆*P* and the current *I* for (**a**) Channel 1; (**b**) Channel 2; and (**c**) Channel 3.

Figure 5 describes the relationship between the output signal ∆*P* and the current *I* when *K* = 2, *K* = 6, and the basic structure [1]. Figure 5 uses the mean squared error to display the measurement data, which includes six consecutive experiments using the same configuration. The precision and accuracy of the experimental results were confirmed by error analyses. Figure 5a,b shows the results of Channels 1 and 2, respectively, with the direct current ranging from 0 to 1000 A. Figure 5c shows the results of channel 3 with the direct current ranging from 0 to 2000 A. Furthermore, the figures compare *K* = 2, *K* = 6 and the basic structure. For *K* = 2, *K* = 6, and the basic structure, the results showed that ∆*P* was approximately linear with *I*. For Channels 1 and 2, the current sensitivity of *K* = 6 was approximately three times as high as when *K* = 2, and approximately six times as high as the basic structure. Furthermore, for the Channel 3 sensor, the current sensitivity when *K* = 6 was twice as high as that when *K* = 2 and five times as high as the basic structure. It can be described by the chi-squared (Chi^2^) test for the goodness of the measurements fit for the three channels when *K* = 2, *K* = 6, and the basic structure. For Channel 1 the Chi^2^ test was 0.98874, 0.99759, and 0.99632 when *K* = 2, *K* = 6, and the basic structure, respectively. For Channel 2, the Chi^2^ test was 0.99482, 0.99480, and 0.99418 when *K* = 2, *K* = 6, and the basic structure, respectively. For Channel 3, the Chi^2^ test was 0.99813, 0.98778, and 0.99907 when *K* = 2, *K* = 6, and the basic structure, respectively. The three channels had good linearity. In fact, the current sensitivity was dependent on the phase shift caused by the circular and linear birefringence. Considering that the three fiber sensor heads had different values of circular and linear birefringence, the three channels had different current sensitivities. Every channel was independently affected by the current at the measuring point; hence, they need to be calibrated separately. All experimental results showed good linear responses of their respective channels, and the results indicate the feasibility of the hybrid structure to expand the measurement area efficiently.

## 5. Conclusions

A hybrid-structure all-fiber current sensor that can obtain more information via time-division multiplexing was demonstrated in this paper. The sensor using the hybrid structure could obtain more information for the multichannel. The channels share one pulsed laser, one detector, and one digital signal processing system. The results showed that currents could be simultaneously measured at different points in a system or at different systems with effectively no added cost. The hybrid-structure all-fiber current sensor exhibited a good light isolation effect and good light utilization efficiency. In the experiment, the three-channel fiber current sensor showed an excellent linear independence of the three channels. This allowed us to design a hybrid-structure all-fiber current sensor which has advantages of better flexibility, smaller size, and lower cost.
